# Apoptosis Maintains Oocyte Quality in Aging *Caenorhabditis elegans* Females

**DOI:** 10.1371/journal.pgen.1000295

**Published:** 2008-12-05

**Authors:** Sara Andux, Ronald E. Ellis

**Affiliations:** Department of Molecular Biology, UMDNJ School of Osteopathic Medicine, Stratford, New Jersey, United States of America; Huntsman Cancer Institute, United States of America

## Abstract

In women, oocytes arrest development at the end of prophase of meiosis I and remain quiescent for years. Over time, the quality and quantity of these oocytes decreases, resulting in fewer pregnancies and an increased occurrence of birth defects. We used the nematode *Caenorhabditis elegans* to study how oocyte quality is regulated during aging. To assay quality, we determine the fraction of oocytes that produce viable eggs after fertilization. Our results show that oocyte quality declines in aging nematodes, as in humans. This decline affects oocytes arrested in late prophase, waiting for a signal to mature, and also oocytes that develop later in life. Furthermore, mutations that block all cell deaths result in a severe, early decline in oocyte quality, and this effect increases with age. However, mutations that block only somatic cell deaths or DNA-damage–induced deaths do not lower oocyte quality. Two lines of evidence imply that most developmentally programmed germ cell deaths promote the proper allocation of resources among oocytes, rather than eliminate oocytes with damaged chromosomes. First, oocyte quality is lowered by mutations that do not prevent germ cell deaths but do block the engulfment and recycling of cell corpses. Second, the decrease in quality caused by apoptosis mutants is mirrored by a decrease in the size of many mature oocytes. We conclude that competition for resources is a serious problem in aging germ lines, and that apoptosis helps alleviate this problem.

## Author Summary

As women age, the quality of their oocytes declines, causing the eggs they make to have a higher chance of producing a miscarriage or a child with birth defects. We used the roundworm *C. elegans* to study this problem. We show that oocyte quality declines in these small animals during aging, much as in mammals. Furthermore, our results show that the programmed deaths of many developing oocytes help maintain the quality of the oocytes that survive, resulting in better eggs. These cell deaths appear to regulate the way resources are allocated in the aging germ line. Since many oocytes die in humans as well as in nematodes, our studies point to the possibility of improving oocyte quality by manipulating cell death in the germ line.

## Introduction

As women age, the quality and quantity of their oocytes decline, resulting in a decreased chance of becoming pregnant and an increased chance of having a child with birth defects [1],[2]. A major cause of this decline is the increasing fraction of oocytes with chromosomal abnormalities, such as those that cause Down's syndrome. These abnormalities are caused, at least in part, by defects in recombination and chromosome cohesion during meiosis [3],[4]. In theory, the accumulation of other types of mutations, a decreased ability to eliminate defective oocytes, or fewer resources to nurture developing oocytes might also contribute to the decline in oocyte quality. While this decline in quality is occurring, many other oocytes are undergoing apoptosis [5]. It is not known what role these apoptotic deaths play in oogenesis and the maintenance of oocyte quality.

The nematode *Caenorhabditis elegans* is one of the leading models for studying germ cell development [6] and apoptosis [7]. The *XX* animals are self-fertile hermaphrodites and the *XO* animals are males. At 15°C, the first 60–80 germ cells in hermaphrodites develop as spermatocytes, resulting in 240–320 sperm, and subsequent germ cells develop as oocytes. In other respects, the hermaphrodites are similar to females from related species; in particular, they have female gonads, which consist of two symmetrical U-shaped tubes connected by a central uterus. The distal end of each tube contains a stem cell niche, where germ cells proliferate under the influence of the Distal Tip Cell [6]. As they move farther down the tube, germ cells enter the transition zone and begin meiosis. Soon afterward, the developing germ cells appear to pause or arrest *during* the pachytene stage of Prophase I. To complete pachytene, they require a signal from the Ras/MAPK pathway [8],[9]. At this point, many germ cells begin to increase in size [8], but more than half of the developing oocytes undergo apoptosis [10],[11]. The remaining germ cells move into the proximal gonad, progress to diakinesis of prophase I, and arrest until activated by Major Sperm Protein to begin meiotic maturation [12]. After maturation, oocytes are fertilized and ovulated. They quickly complete meiosis, acquire an egg shell, begin embryogenesis, and are laid.

In hermaphrodites, about half of all germ cells [10],[11] and 10% of all somatic cells [13],[14] undergo apoptosis. All of these deaths are controlled by a common genetic pathway [7]—CED-3 is an executioner caspase that causes apoptosis, the Apaf-1 homolog CED-4 binds to and activates CED-3, and the Bcl-2 homolog CED-9 binds to and antagonizes CED-4. Additional genes mediate the engulfment and removal of dying cells, but do not cause programmed cell death [7]. Finally, the BH3-containing protein EGL-1 inactivates CED-9 in the appropriate somatic cells, causing the release of CED-4, which initiates apoptosis. Although many oocytes die, apoptosis does not occur during spermatogenesis and is not seen in the male germ line [10].

Physiological germ cell deaths require both *ced-3* and *ced-4*, but are not affected by mutations in *egl-1* or by the *ced-9(gf)* mutation [10], which prevents EGL-1 from causing the release of CED-4 [15],[16]. Although the majority of oocytes die in wildtype animals, both *ced-3* and *ced-4* mutants reproduce in large numbers even though no germ cell deaths occur. Thus the importance of these deaths has been unclear.

Germ cells can also undergo apoptosis in response to DNA-damage [17]. These deaths require CEP-1 (*C. elegans* p53 homolog), which acts through *egl-1* and *ced-13* to regulate *ced-9* activity [18],[19]. Loss-of-function mutations in any of these genes, as well as the *ced-9(n1950*gf*)* mutation, prevent cell death in response to DNA damage, but have no effect on physiological germ cell deaths. Unsynapsed chromosomes also initiate apoptosis in germ cells through a process that does not require *cep-1* but does use the AAA ATPase PCH-2 [20]. Finally, other kinds of stress induce germ cell deaths that occur independently of *cep-1* [21],[22].

Recent studies have shown that nematodes show an age-related decline in the number of progeny they produce [23], but it is not clear what factors underlie this decline. In this paper, we focus on how oocyte quality is influenced by aging, and how apoptosis affects oocyte quality. Since hermaphrodites produce sperm, each new oocyte is fertilized soon after it matures, which makes it difficult to study changes during aging. Thus, we have been using *fog-2(q71)* females, which do not make sperm [24], causing the oocytes to accumulate or “stack” within the gonads of virgin females. By delaying fertilization, we could study the quality of oocytes produced at different ages. We show that nematode oocytes decline in quality during aging, much as in mammals. Furthermore, we demonstrate that physiological germ cell deaths play a key role in maintaining oocyte quality, and that they function by promoting the efficient allocation of resources into developing oocytes.

## Methods

### Worm Strains and Culture Conditions

Nematodes were handled using standard methods [25]. Animals were maintained on NG plates at 15°C (NG Agar: 6 g NaCl, 9 g KH_2_PO_4,_ 1.5 g K_2_HPO_4_, 12 g tryptone, 60 g Agar, and 1 ml cholesterol in ethanol (15 mg/ml) are added to 3 L dH_2_O and autoclaved). To score progeny, the animals were raised on Low Growth NG plates (as above, without tryptone) at 15°C so that the bacterial lawn remained thin enough to allow accurate counts of eggs and larvae. To age the animals, females were first collected in the late L4 stage and checked for adulthood approximately 5 hours later. Those that had reached adulthood were then aged for 24 hours, 72 hours, or 144 hours for further assays. Females still in L4 were used for zero hour assays.

All strains were derived from the wild-type Bristol strain N2, and included the *fog-2(q71)* mutation to prevent sperm production in hermaphrodites, unless otherwise indicated. The alleles used in this study were: LG I: *ced-1(e1735)* [26], *fog-1(q250)* [27]; LG III: *ced-4(n1162), ced-4(n2274)* [28], *ced-6(n1813)* [29], *ced-9(n1950)* [30]; LG IV: *ced-3(n718), ced-3(n2439), ced-3(n2921)* [31],[32]; LG V: *fog-2(q71)* [24], *egl-1(n1084n3082)* [33] *egl-1(n3330)* (B. Conradt and H.R. Horvitz, personal communication).

### Time Course Assays

Females were allowed to mate with 5 males for 10–14 hours before the males were removed. The females were then transferred to new plates every 12 hours until egg production ceased or until the female was unable to continue laying eggs. Eggs and larvae were counted at 0 hours, 12 hours, 24 hours, and 48 hours after the female had been transferred. Eggs that had not hatched by 48 hours were scored as dead; larvae that had not progressed past the L1 stage by 48 hours were scored as terminally arrested in development. We considered eggs less than approximately 1/3 normal size to be inviable and did not include them in our assays, but did note how frequently they occurred. Eggs greater than 1/3 normal size but still undersized were included in all of our assays, and their frequency was also noted. Line graphs only include time points for which at least 10 eggs for each age and genotype were available.

### Oocyte Assays and Microscopy

To measure the effects of aging on specific oocytes, females were aged as described, anesthetized and mounted on slides [13] immediately prior to mating. Differential interference contrast microscopy was used to examine the germlines and count the number of full-grown oocytes stacked in the female gonad. The females were recovered and crossed with males for 12 hours, or until egg production began. Males were then removed and the females were transferred to new plates every 2–3 hours until all stacked oocytes had been fertilized and laid.

## Results

### Oocyte Quality Decreases with Maternal Age

**Figure 1:**
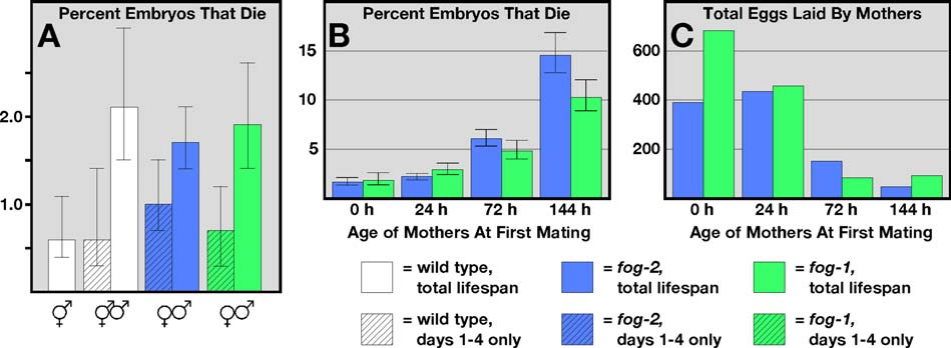
Oocyte quality decreases with maternal age. (A) Percent of eggs that died before hatching. The mothers were wildtype hermaphrodites (white), *fog-2(q71)* females (blue), and *fog-1(q250)* females (green). Solid bars represent the fraction of all eggs that died throughout the entire reproductive span; shaded bars represent the fraction of eggs that died during the first four days of the reproductive span. Error bars represent 95% confidence intervals. (B) Percent of eggs that died before hatching, produced by females mated at the specified ages. (C) Total eggs produced by females mated at the specified ages.

Nematode hermaphrodites reproduce early in adulthood and quickly exhaust their supply of self-sperm. To see if their oocytes change during aging, we began studying mated females, which have a larger supply of sperm and reproduce for a longer time. We found that almost all of the eggs produced by hermaphrodites or mated females during the first four days of adulthood were viable (Figure 1A, Dataset S1). However, viability declined when we assayed eggs made during the entire reproductive lifespan of females or mated hermaphrodites. Furthermore, this result was independent of the mutation we used to induce female development. Since the viability of eggs reflects the quality of the oocytes that produced them, we conclude that the quality of oocytes produced later in life is lower than that of earlier ones.

Two simple models could explain this decline in quality: (1) these females might only be able to produce a limited number of high quality oocytes, and all additional oocytes would be inferior, or (2) regardless of the number of oocytes already produced, older mothers might produce oocytes of lower quality than younger mothers. To distinguish between these models, we allowed females to age before crossing them with males. We found that older mothers produced oocytes of significantly lower quality than younger ones (Figure 1B, Dataset S1), and also produced fewer fertilized eggs altogether (Figure 1C, Dataset S1). Thus, the decline in oocyte quality was determined by maternal age, rather than by the absolute number of eggs produced during an animal's lifespan.

In these assays, all of the viable eggs yielded healthy larvae, regardless of the mother's age (>99.5%; data not shown), so the main effect of low oocyte quality was on embryos. Since *fog-2(q71)* and *fog-1(q250)* females gave comparable results, the influence of aging on oocyte quality was independent of genetic background.

### Aging Affects Two Populations of Oocytes

**Figure 2:**
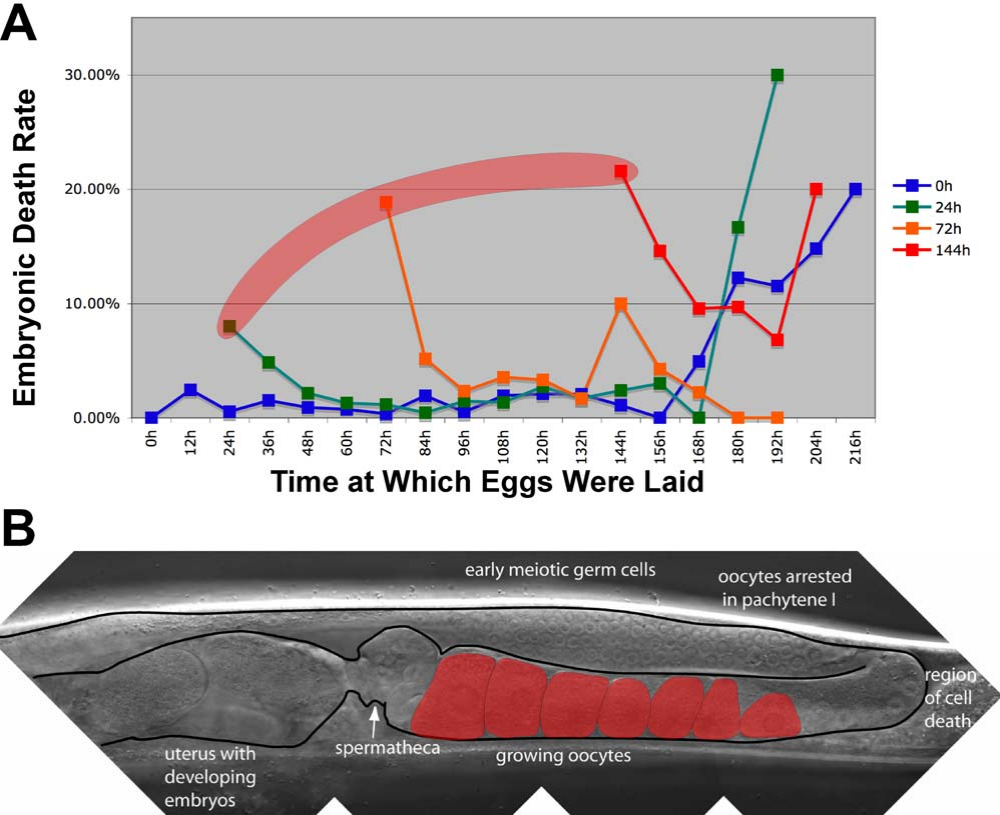
Aging affects two populations of oocytes. (A) Percent of eggs that died before hatching, plotted against the age of the mother when the eggs were laid (in hours after the molt into adulthood). The mothers were virgin *fog-2(q71)* females mated at zero hours (blue), 24 hours (green), 72 hours (orange) and 144 hours (red). The shaded area indicates eggs formed from oocytes that had been arrested in diakinesis. (B) Nomarski photomicrograph of the gonad of a mated female. Arrow indicates the spermatheca. Shaded oocytes are arrested in diakinesis. Anterior is to the left, and ventral is down.

To learn when the effect of maternal age on oocyte quality was most pronounced, we followed groups of eggs laid during 12-hour intervals over a female's entire lifespan, and determined what fraction survived and hatched (Figure 2). We found that two groups of oocytes were of lower quality than the rest: (1) the first oocytes fertilized in aging females, and (2) oocytes that developed more than 6 days after a female had matured, regardless of her age at mating.

To determine if the initial decline in quality was due to ‘stacked’ oocytes that had spent an extended period of time arrested in diakinesis, we counted the number of stacked oocytes in 72-hour females just before mating, and then observed how many produced viable eggs. We found that 12% of the eggs that developed from stacked oocytes died before hatching (17 dead eggs from 146 stacked oocytes), compared with 3% of the eggs produced from oocytes that matured shortly afterward (3 dead eggs from 86 oocytes). Thus, an extended arrest in diakinesis was detrimental to oocyte quality.

### Larval Deaths Caused by Blocking Apoptosis Are Not Due to Defective Oocytes

**Figure 3:**
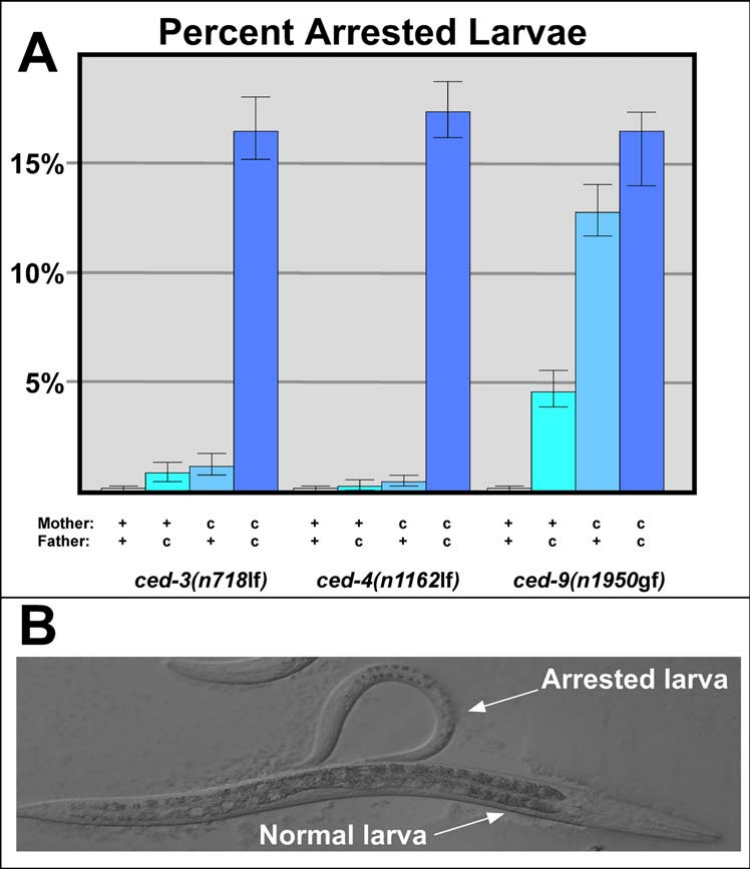
The larval lethality caused by apoptosis mutants is not due to low oocyte quality. (A) Females with (c) or without (+) the indicated mutation were crossed with corresponding males, and their offspring were assayed for growth. Bars indicate the percent of larvae from each cross that remained in the L1 stage after 48 hours. Error bars represent 95% confidence intervals. (B) Nomarski photomicrograph of two *ced-3(n718)* larvae at 48 hours post-fertilization. The animal at the top had permanently arrested at this stage, and eventually died.

Previous studies reported that some *ced-3* larvae arrest development before reaching adulthood [34]. In fact, we found that 16.5% of all *ced-3(n718)* larvae did not develop past the L1 larval stage (Figure 3A, Dataset S1), although the rest grew normally. Because we suspected that this problem might be caused by variations in maternal oocyte quality, we looked at heterozygous offspring produced by crossing *ced-3* females with wild-type males and *vice versa.* Since the offspring from both of these crosses showed less than 1% larval arrest (Figure 3A), the maternal *ced-3* genotype did not cause the lethality. We repeated these crosses using older females and found similar results (data not shown). Further experiments using *ced-3(n2439), ced-3(n2921), ced-4(n1162)*, *ced-4(n2274), egl-1(n1084n3082)* and *egl-1(n3330)* showed the same effect (Figure 3 and data not shown). Finally, analysis of *ced-9(n1950*gf*)* females showed that the severity of the cell death defect correlated with the rate of larval lethality (Figure 3A). The *n1950* mutation is semi-dominant and has a maternal effect [30]. Heterozygous progeny from wild type mothers show low levels of cell survival [30] and low levels of larval arrest (Figure 3A); heterozygous progeny from *n1950* mothers show higher levels of cell survival [30] and higher levels of larval arrest (Figure 3A). Thus, blocking cell death causes some larvae to halt development and eventually die in the L1 stage, but oocyte quality plays no role in this process. We suspect that certain surviving cells occasionally interfere with normal development.

### *ced-3* and *ced-4* Oocytes Are of Lower Quality than Wild-Type Oocytes

**Figure 4:**
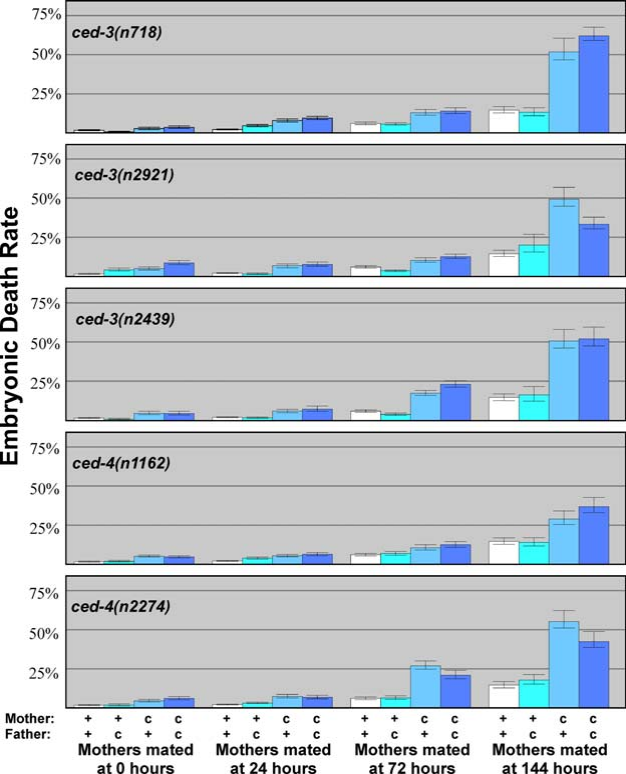
Blocking germ cell death lowers oocyte quality in aging females. Virgin females with (c) or without (+) the indicated mutation were aged and crossed with males of the indicated genotype. Bars represent the percent of eggs that died throughout the entire reproductive span. The first bar (white) in each age group represents control data from Figure 1B. Error bars represent 95% confidence intervals.

During these studies, we found that *ced-3(n718)* eggs died before hatching more frequently than wildtype eggs (Figure 4, Dataset S1). To see if this effect was due to decreased oocyte quality in the *ced-3* mothers, we again looked at heterozygous offspring. All *ced-3* mothers produced more dead eggs than did wildtype mothers, irrespective of the offspring's genotype (Figure 4), indicating that this effect was indeed maternal, and thus reflected a decrease in oocyte quality. Furthermore, the fraction of eggs from *ced-3* mothers that died increased with maternal age, and this increase was more dramatic in *ced-3* mothers than in wildtype mothers (Figure 4). We repeated these experiments using *ced-3(n2439)* and *ced-3(n2921)* females and observed the same effect (Figure 4), so it was caused by a decrease in *ced-3* activity, rather than by a linked mutation. This age-related decline is specific to the germline, since *ced-3(lf)* animals have normal lifespans [35],[36].

We also determined the number of eggs laid by *ced-3* females. Since no germ cells were being eliminated by apoptosis, we had expected that *ced-3* females might produce more eggs than the wild-type. We found that the total number of eggs they laid was substantially lower, particularly as the females aged (the average brood size of all 72 hour- and 144 hour-aged *ced-3* mothers was 126 and 27 eggs respectively, compared with 180 and 65 eggs for wildtype mothers). However, we also saw an increase in the number of tiny, egg-like objects laid by these mothers, which suggested that *ced-3* mutants had difficulty producing full-sized oocytes (the average number of small egg-like objects for all 72 hour- and 144 hour-aged *ced-3* mothers was 15 in both cases, compared with 2 and 8 respectively for wildtype mothers).

Finally, we asked if the effect on oocyte quality we observed in *ced-3* females was exclusive to *ced-3,* or if other apoptotic genes were involved. Thus, we repeated our experiments using two alleles of *ced-4, n1162* and *n2274*. We found that all *ced-4* mothers produced more dead eggs than did wildtype mothers (Figure 4), implying that apoptosis itself is needed to maintain oocyte quality. As with *ced-3*, the *ced-4* effect increased with age, causing a severe, early decline in oocyte quality, as well as lower total numbers of eggs and increased numbers of tiny, egg-like objects (data not shown).

### Wildtype *ced-3* and *ced-4* Activities Prevent a Premature Decline in Oocyte Quality

**Figure 5:**
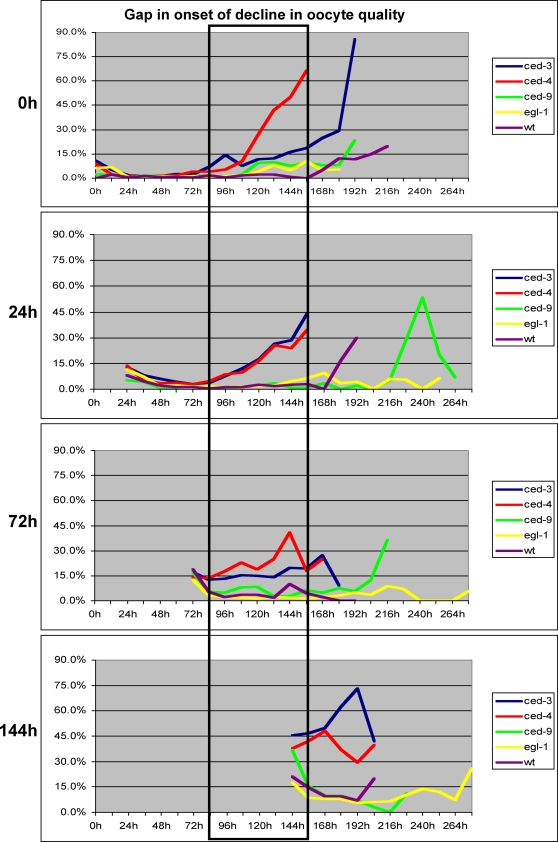
Mutations in *ced-3* or *ced-4* cause a premature decline in oocyte quality. Percent of eggs laid in 12 hour intervals that died. We plotted an average of all *ced-3(lf)* females (blue), all *ced-4(lf)* females (red), *ced-9(n1950*gf*)* females (green), all *egl-1(lf)* females (yellow) and wildtype females (purple). The females were first mated at zero hours, 24 hours, 72 hours or 144 hours after sexual maturation. The boxed area indicates the age when oocyte quality from *ced-3(lf)* and *ced-4(lf)* mothers begins to decline. Wildtype data is from Figure 2A. A paired T test gives a two-tail P value <0.01 for both *ced-3(lf)* and *ced-4(lf)* compared with the wild type, at all ages and across all time points. By contrast, the two tail P value >0.1 for both *egl-1(lf)* and *ced-9(gf)* compared with the wild type, at all ages and across all time points, except for *ced-9(gf)* at 0h (P = 0.03) and *egl-1(lf)* at 144h (P = 0.06).

To determine when these mutants were producing defective oocytes, we monitored embryonic lethality in groups of eggs laid at 12-hour intervals over the course of the females' lifespan. We found that oocyte quality in both *ced-3* and *ced-4* mothers began to deteriorate 3–4 days after sexual maturation (Figure 5, boxed areas), compared with 6–7 days for wild-type mothers. Thus, blocking all cell deaths strongly influenced the quality of newly formed oocytes in older females. We also observed a modest decrease in viability among eggs produced from oocytes that had spent a prolonged period of time in diakinesis for both *ced-3* and *ced-4* mothers (Figure 5).

### Neither *ced-9(n1950*gf*)* Nor *egl-1(lf)* Mutations Decrease Oocyte Quality

**Figure 6:**
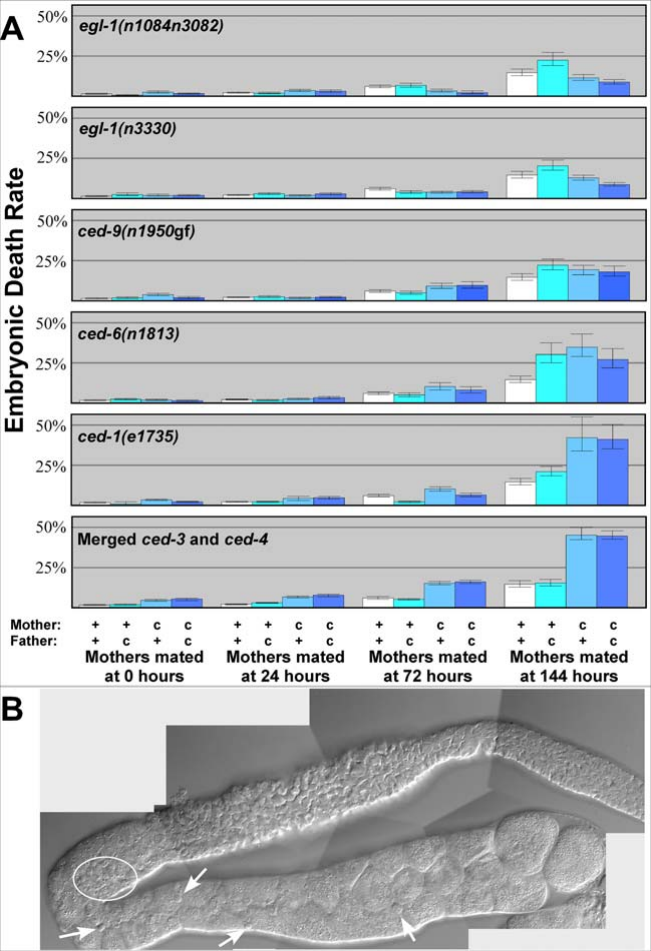
Oocyte quality depends on the death and removal of germ cells. (A) Virgin females with (c) or without (+) the indicated mutation were aged as indicated and crossed with males of the indicated genotype. Bars represent the percent of eggs that died throughout the entire reproductive span. Error bars represent 95% confidence intervals. Wildtype data from Figure 2A, and combined *ced-3* and *ced-4* data from Figure 4 are provided for comparison. (B) Nomarski photomicrograph of an extruded gonad from a 144 hour-old *ced-1(e1735)* female. Arrows indicate individual corpses scattered among maturing oocytes; the circle marks a cluster of corpses in the region where germ cells exit from the pachytene stage.

Mutations in *ced-3* and *ced-4* prevent cell deaths in both the soma and the germline. However, the gain-of-function mutation *ced-9(n1950)* blocks somatic cell deaths, but does not affect physiological germ cell deaths [10],[30]. To see if the decline in oocyte quality we observed in *ced-3* and *ced-4* mutants was caused by the lack of cell death in the soma, we studied eggs laid by *ced-9(n1950)* females. We found that their oocytes were, on average, as healthy as those from wildtype females for all time points and for mothers of all ages (Figures 5, 6A). Thus, somatic cell deaths are not required to maintain oocyte quality.

To confirm these results, we also studied two loss-of-function mutations in *egl-1,* a gene that negatively regulates *ced-9.* These *egl-1* mutations also block somatic cell deaths, but do not affect physiological germ cell deaths [10]. As with *ced-9(gf),* the *egl-1(lf)* mutants produced oocytes that were at least as healthy as those from wild-type females (Figures 5, 6A, Dataset S1). We conclude that blocking somatic cell deaths does not influence oocyte quality.

Since the mutations in *ced-9* and *egl-1* also prevent germ cells from undergoing cell death in response to DNA damage [37], our results imply that apoptosis does not normally maintain quality by eliminating oocytes that contain damaged DNA. Instead, high oocyte quality is maintained by the physiological germ cell deaths that occur in aging females.

### Mutations That Prevent the Engulfment of Cell Corpses Lower Oocyte Quality

Two models could explain how germ cell deaths maintain oocyte quality. In the first, apoptosis eliminates defective oocytes from the germ line. In the second, apoptosis modulates the number of developing oocytes to help allocate resources properly. To distinguish between these models, we looked at the effect of mutations that do not block cell death, but do prevent the engulfment and metabolism of cell corpses. We found that mutations in both *ced-1* and *ced-6* decrease the quality of oocytes, although not as severely as do mutations in *ced-3* or *ced-4,* which block cell death altogether (Figure 6A, Dataset S1). When we examined these mutants, we found that their germ lines were often less well-organized in older females (Figure 6B), although not as severely compromised as in *ced-3* mutants. Since virgin *ced-1* females had an average of 16 corpses per gonad arm at 72 hours (n = 14) and an average of 21 corpses at 144 hours (n = 30), cell death is still ongoing in those worms. Thus, physiological germ cell deaths appear to maintain oocyte quality by regulating the allocation of resources in the aging germ line. These deaths might act directly by decreasing competition between oocytes, or indirectly by nourishing the somatic gonad, which engulfs each corpse and regulates meiotic maturation in surviving oocytes.

### The Decrease in Oocyte Quality Correlates with a Decrease in Oocyte Size

**Figure 7:**
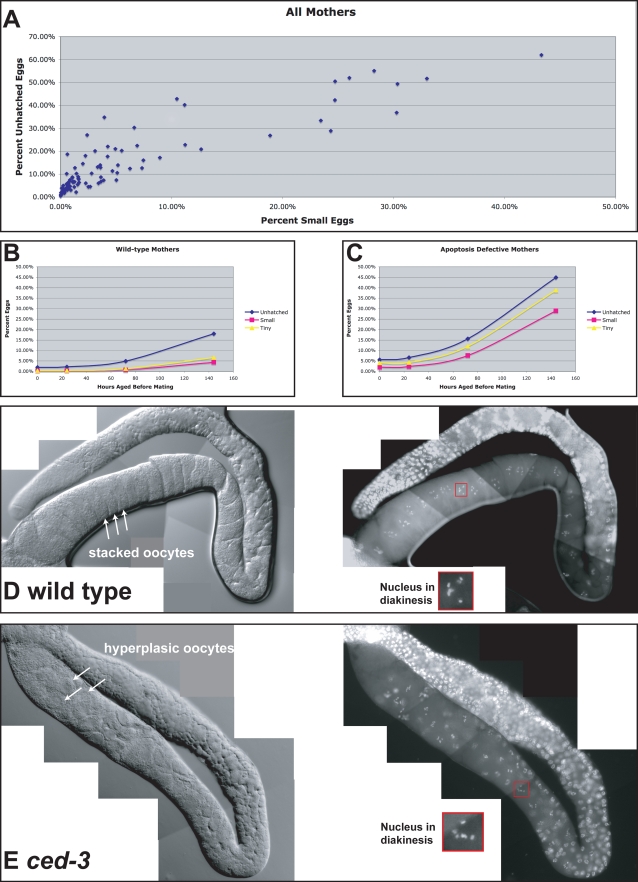
Decreased oocyte quality correlates with decreased oocyte size. (A) Scatter plot comparing percent embryonic death with percent of small eggs. Each point represents data for females from one set of crosses that were mated at a single age. (B) Percent embryonic death (blue), percent of small eggs laid (pink), and percent tiny, egg-like objects produced (yellow), plotted against the mother's age at first mating. Graph presents combined data for all wildtype mothers, regardless of the genotype of the father. (C) As in B, but the graph presents combined data for all *ced-3* and *ced-4* mothers, regardless of the genotype of the father. (D) Photomicrographs of the extruded gonad from a wild-type virgin that was aged for 144 hours after maturation. The left panel shows a Nomarski image, the right panel shows the same gonad stained with DAPI, and the inset shows a typical nucleus in diakinesis, which we used as a marker for oocytes in the last phase of development. (E) Photomicrographs of the extruded gonad from a *ced-3(n718)* virgin female that was aged for 144 hours after maturation. Notice the larger number and smaller size of oocytes in the last phase of development.

Mutations in *ced-3* or *ced-4* cause germline hyperplasia during aging, resulting in more germ cells but fewer fully grown oocytes [10]. If additional resources are directed to these extra cells, this defect could lead to the production of small eggs that lack the resources needed for embryogenesis. Thus, we noted each time we observed eggs that were smaller than normal. If germ cell deaths were indeed needed to allocate resources in the germ line, low oocyte quality might correlate with the frequency of small eggs. When we plotted the relationship between oocyte quality and the frequency of small eggs, using data points for females of each age and genotype we had examined, we observed a roughly linear relationship between these traits, with a correlation coefficient of 0.90. (Figure 7A). In this compound data set (n = 216,875 eggs), 6.0% of all eggs died before hatching, but we only scored 1.8% of the eggs as small. Some of the eggs that appeared normal might have had more subtle differences in size or composition.

To determine if oocyte volume changes during aging, we graphed the frequency of small eggs among the progeny of wild-type mothers first mated at 0, 24, 72 or 144 hours (Figure 7B), and of apoptosis defective mothers first mated at each of these times (Figure 7C). For each graph, we also plotted the frequency of eggs that died before hatching. Finally, we plotted the frequency of tiny, egg-like objects we had observed in our studies but not included in our calculations of oocyte quality, since they appeared too small to be viable. We found correlation coefficients of greater than 0.997 for each of these markers of oocyte size with the quality of oocytes made by these females. In theory, small eggs could be produced either by the fertilization of small oocytes, or by the breakdown of larger oocytes. However, at the same time that the frequency of small eggs produced by *ced-3* or *ced-4* mutants was increasing (Figure 7C), the number of maturing oocytes in their gonads was also increasing [10] and the average size of these oocytes was decreasing. In the wild type, the stacked oocytes each occupied an entire slice of the gonad (Figure 7D), whereas in *ced-3* mutants, the oocytes were smaller and lay along the surface of the germline, with additional oocytes located underneath in the same region (Figure 7E). In fact, we observed an average of 47 oocytes in diakinesis in *ced-3* mutants (n = 6 gonad arms) but only 20 in the wild-type (n = 6 gonad arms), even though these cells were distributed over volumes that were equivalent in size. Thus, we conclude that aging animals have difficulty providing sufficient resources to nurture full-sized oocytes, and that defects in apoptosis aggravate this problem, contributing to the decline in oocyte quality.

## Discussion

### Nematode Oocyte Quality Declines with Age

In this paper, we show that oocyte quality declines in aging nematodes. This characteristic has not been described previously, because of the nature of hermaphrodite reproduction. In *C. elegans,* a wild-type hermaphrodite can produce about 2000 germ cells during its lifetime. However, the first 60–80 germ cells develop into 240–320 sperm, which fertilize each new oocyte soon after it is fully grown. Thus, hermaphrodites tend to reproduce exclusively at young ages, and over 99% of their self-fertilized eggs survive and hatch into healthy larvae. By working with female nematodes, we found that the oocytes produced and fertilized later in life, after a hermaphrodite's normal sperm supply would have been depleted, are of lower quality than oocytes produced at a younger age. Thus, aging nematodes show a decline in fertility, as occurs with humans and many other animals. This similarity makes *C. elegans* an attractive model for studying how oocyte quality is controlled during aging.

The age-related decline of oocyte quality and quantity in humans is well documented [1],[2],[38],[39],[40],[41]. By the time a female reaches puberty, the millions of oocytes she was born with will have dwindled to about 300,000, with hundreds vanishing each month thereafter [2]. This rate of loss increases dramatically when a woman reaches about 37 years of age and continues until menopause, at which point less than 1000 follicles remain [40]. Oocyte quality decreases in parallel with a decrease in the oocyte population, resulting in an increased rate of defective mature oocytes as a woman ages. As a result, an older female is less likely to become pregnant than a younger female, and those that do become pregnant have a higher risk of having a child with birth defects, or losing the pregnancy altogether by miscarriage.

Our studies show that the aging process affects two populations of oocytes in nematodes: (1) oocytes that were arrested in diakinesis while awaiting a signal to mature and be fertilized, and (2) developing oocytes in older mothers. Rather than compare human and nematode oocytes based only on their progression through meiosis, we suggest that the most important features to consider involve their underlying biology. In particular, oocytes in the first group have reached a mature size and are no longer susceptible to cell death [10], so they might not be comparable to any stage of human oogenesis. However, the second group contains many cells that are arrested in prophase of meiosis I, awaiting a MAPK signal to grow or initiate apoptosis. Thus, they might be analogous to the vast population of human primordial follicles, which are also arrested in prophase of meiosis I, awaiting signals to develop into primary follicles or undergo cell death [42],[43].

### Oogenesis Shares Common Steps in Nematodes and Mammals

Do similarities in the aging process reflect similar biological causes? Although they differ in many ways, nematode and mammalian oogenesis share several common steps. First, germ cells in both groups proliferate in a stem cell niche created by the somatic gonad [6],[44]. Second, during early meiosis developing oocytes in both groups share cytoplasm—in the early stages of human follicle development, oocyte nuclei cluster together but are not separated by membrane boundaries [44],[45],[46], and in nematodes young oocytes are part of a large syncytium [6]. Third, oocytes in both species make a major transition near the end of pachytene. In humans, folliculogenesis begins after oocytes exit from pachytene of meiosis I and form complete cell boundaries, each surrounded by somatic granulosa cells [47], and in nematodes, oocytes exit from pachytene of meiosis I and form contacts with a new set of somatic gonadal sheath cells [48]. Finally, in both species oocytes arrest near the end of prophase in meiosis I, and wait for a signal to mature. Although these steps display many species-specific peculiarities, the underlying pathways that regulate somatic/germ cell interactions, that control the progression through meiosis, and that detect and respond to problems could be similar.

### Does Paternal Age Influence Embryonic Survival?

Many studies done in mammalian systems suggest that the age of the father might also influence the viability of a developing embryo [49],[50],[51]. Specifically, offspring from older fathers are at higher risk of developing autism spectrum disorders or other neurodevelopmental problems [52],[53]. Two observations suggest that paternal age is relatively unimportant in worms. First, we see no difference in embryo viability between a hermaphrodites' normal brood of 240–320 eggs and the first 240–320 eggs laid by females mated at sexual maturation (Figure 1A). Second, we performed a pilot study using young adult (zero hour) females mated to males aged 0 hours, 144 hours, or 216 hours past sexual maturation and found no differences in embryonic lethality (1.9%, 2.6% and 1.6% respectively) or normal larval development (>99% in all cases).

In nematodes, a hermaphrodite's health is harmed by mating with males [54],[55]; in theory, this effect could influence oocyte quality. Nonetheless, we suspect it is unimportant, since hermaphrodites and young females mated at sexual maturation produce oocytes of similar quality for at least four days (Figure 1A).

Other studies also documented an age-related decline in progeny production [23]. Does this decline in the number of eggs produced by an aging nematode mirror the decrease in oocyte numbers seen in aging mammals? We suspect not, since nematodes continue to produce germ cells throughout their adult life, and some mature oocytes are visible even in very old animals that no longer lay eggs. Thus, the decline in the number of fertilized eggs is a complex phenomenon that does not merely reflect the number of oocytes.

### Apoptosis Plays a Significant Role in Oogenesis

Programmed cell death plays a major role in oogenesis in most animals [44],[56],[57]. Indeed, this is the predominant fate of developing oocytes in both humans and nematodes. In humans, approximately 7 million oocytes are produced during embryogenesis, but most of these die, and only about 400 follicles are normally ovulated during a woman's lifetime [47]. Similarly, a hermaphrodite nematode produces about 1700 oocytes over its lifetime, yet fewer than half survive to mature [10],[11], and a normal hermaphrodite only lays about 300 eggs [58]. Furthermore, in both groups a massive wave of cell death occurs around the time that oocytes exit from pachytene in prophase of meiosis I [10],[44]. In mammals, a second wave of cell death affects the follicles that contain oocytes arrested in diplotene.

We have shown that apoptosis plays a critical role in maintaining oocyte quality, but how it does so remains uncertain. Three popular theories explain how germ cells might be selected to die [57]: (1) the unfit oocyte theory, in which apoptosis removes defective oocytes from the pool, allowing only healthy oocytes to mature, (2) the nurse cell theory, in which some germ cells are selected to nourish maturing oocytes and later undergo apoptosis, as in *Drosophila* [59], and (3) the neglected oocyte theory, which maintains that nutrients and other factors are in short supply in the germline, requiring some cells to die so that others have the resources to develop properly. These theories are not mutually exclusive, and germ cells might die for any one or a combination of these reasons. We propose that in *C. elegans,* most oocyte deaths function to help redistribute resources in the germ line.

### Apoptosis Can Eliminate Oocytes with Defective Chromosomes

One potential role for apoptosis is the elimination of oocytes with certain types of genetic defects. In particular, unsynapsed chromosomes can trigger apoptosis in human oocytes [60] and in nematodes [20]. In addition, DNA-damage can act through an independent pathway to induce apoptosis in both species [37],[61]. Since maternal age is the most important factor in trisomy formation [1],[62], and spindles in the meiotic oocytes of older females are less organized and the chromosomes are less firmly attached [2], these types of death might maintain quality during aging by eliminating oocytes with chromosomal abnormalities.

**Figure 8:**
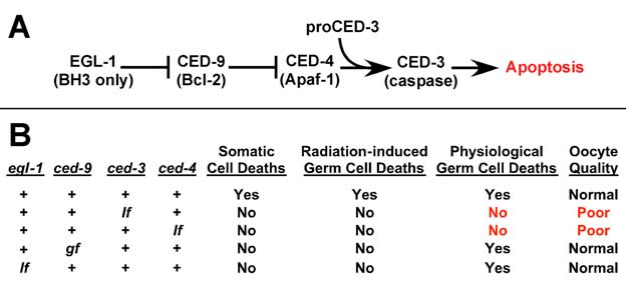
Regulation of oocyte quality by apoptosis. (A) The genetic pathway that regulates apoptosis in *C. elegans.* Arrows indicate activation and “—|” indicates inhibition. Mammalian homologs are indicated in parentheses. (B) Table summarizing the effect of mutations in each of the genes on cell death and on oocyte quality. “+” indicates wildtype function of each gene, “*lf”* and *“gf”* indicate loss and gain of function mutations, respectively.

At least half of the developing oocytes die in nematodes [10]. If germ cell deaths removed only oocytes with damaged chromosomes, then preventing these deaths should result in a high rate of embryonic lethality. However, only 12% of eggs produced by *ced-3* or *ced-4* mothers died before hatching (n = 46,678 eggs). Furthermore, using *ced-9(n1950*gf*)* or *egl-1(lf)* mutations to block germ-cell deaths induced by DNA damage did not lower oocyte quality (Figures 6,8). Thus, we suspect that the numerous physiological germ cell deaths in these animals serve another function.

### Apoptosis Can Eliminate Oocytes Functioning as Nurse Cells

The key feature of nurse cells in *Drosophila* is that they are set aside from birth to produce materials for a developing oocyte, and then eventually undergo apoptosis. Similarly, in humans, maturing follicles contain numerous somatic cells which nourish the developing oocyte until its fate is determined, and many of these cells die [2]. If nematodes used immature oocytes as nurse cells, then blocking cell death should cause a population of these nurse cells to accumulate. To date, no one has reported a surviving population of nurse cells in *ced-3* or *ced-4* mutants [10]. Instead, our results show that the first 100–300 oocytes produced by young *ced-3* or *ced-4* females produce healthy eggs, extending previous observations by Gumienny *et al.* [10]. Thus, we propose that all germ cells in pachytene have an equal potential to develop into mature oocytes, and that none are specifically designated as nurse cells. This model agrees with studies showing that all germ cells in pachytene contribute cytoplasm to developing oocytes [63].

### Apoptosis Can Reduce Competition among Oocytes

We propose that apoptosis helps allocate resources among developing oocytes, with some surviving and growing, and others dying and being recycled. This model rests on the following observations: (1) Oocytes produced by aging *ced-3* or *ced-4* females, which lack all cell deaths, had the poorest quality of any group we studied (Figure 4). (2) These *ced-3* or *ced-4* mothers also produced more small oocytes than the wild type (Figure 7). (3) These problems became more severe as the females aged. (Figures. 5, 7). (4) Oocytes produced by aging *ced-1* and *ced-6* females, which cannot engulf and recycle cell corpses, were also of lower quality than wild-type oocytes (Figure 5). We infer that in the absence of germ cell deaths, there is a premature depletion of resources caused by too many competing oocytes. When germ cells undergo apoptosis but are not engulfed and recycled, a smaller depletion of resources occurs.

Another result supports this model—the quality of the oocytes arrested in diakinesis is lower in *ced-3* or *ced-4* mutants than in the wild type. This decline in quality cannot be due to defects in the oocytes themselves, since these oocytes are of higher quality if fertilized immediately. (Compare *ced-3* data for females mated at zero hours with that for females mated at older ages). Instead the quality of these oocytes declines over time, and declines faster in *ced-3* or *ced-4* mutants than in the wild type. Thus, we suspect that a competition for resources in the *ced(lf)* mutants affects the ability of the aging germ line to nurture and maintain arrested oocytes. Indeed, there are often smaller oocytes interspersed with full-grown oocytes in the proximal gonads of older *ced-3* and *ced-4* females (Figure 7 and data not shown).

Although physiological germ cell death plays a major role in maintaining oocyte quality, other factors could be involved. Stresses such as food deprivation [21],[64], pathogen infection [65], and exposure to toxins [21] can trigger germ cell death by acting through CED-9 [10]. These signals might maintain oocyte quality when animals are raised in adverse conditions.

### Does Oocyte Quality Influence Fertilization?

In our studies, we found that older *ced-3* or *ced-4* mutants stopped producing fertilized eggs at a younger age than the wild-type, even though they continued to produce oocytes. This result suggests that some mechanism regulates the ability of developing oocytes to mature and be fertilized, and that this mechanism plays a major role when quality is declining. If so, altering oocyte quality might also influence an animal's overall fertility. One possibility is that some characteristic of oocytes that directly reflects quality determines if an oocyte is able to mature and be fertilized. Alternatively, the accumulation of extra oocytes in these mutants might interfere with the normal rhythm of maturation and fertilization.

### Can Genetic Manipulations Improve Oocyte Quality and Reproduction during Aging?

We have shown that oocyte quality declines in aging nematodes, as it does in humans, and that apoptosis prevents a premature decline in quality. Could some mutations actually improve oocyte quality and reproductive success? We were surprised to find that both of our *egl-1* mutants produce oocytes of higher quality than the wild type in very old animals (Figure 6A). This difference is significant at a 99% confidence level. Furthermore, these *egl-1* animals reproduce for a longer period of time than the wild type or than other *ced* mutants (Figure 5). Mutations in other genes also extend the reproductive span in nematodes [23],[66]. For comparison, studies with mice show that mutations in *Bax,* which block many germ cell deaths, extend the reproductive lifespan of females [67]. However, the analysis of oocyte quality is difficult in mammals, since embryos develop *in utero,* and it is not known how quality changes in *Bax* mice during aging. Nonetheless, it seems likely that some genetic changes can improve reproductive success in older mothers.

We are now studying the relationship between the control of physiological germ cell deaths and the induction of cell death in response to DNA damage. Furthermore, we are exploring the link between these processes and the DAF-2/insulin-like signaling pathway, which is a conserved regulator of lifespan and development in all tissues [68],[69].

## Supporting Information

Dataset S1 Supporting dataset.
(0.13 MB DOC)

## References

[1] Eichenlaub-Ritter U (1998). Genetics of oocyte ageing.. Maturitas.

[2] te Velde ER, Pearson PL (2002). The variability of female reproductive ageing.. Hum Reprod Update.

[3] Hodges CA, Revenkova E, Jessberger R, Hassold TJ, Hunt PA (2005). SMC1beta-deficient female mice provide evidence that cohesins are a missing link in age-related nondisjunction.. Nat Genet.

[4] Lamb NE, Sherman SL, Hassold TJ (2005). Effect of meiotic recombination on the production of aneuploid gametes in humans.. Cytogenet Genome Res.

[5] Morita Y, Tilly JL (1999). Oocyte apoptosis: like sand through an hourglass.. Dev Biol.

[6] Kimble J, Crittenden SL (2007). Controls of germline stem cells, entry into meiosis, and the sperm/oocyte decision in Caenorhabditis elegans.. Annu Rev Cell Dev Biol.

[7] Conradt B, Xue D (2005). Programmed Cell Death. Wormbook..

[8] Church DL, Guan KL, Lambie EJ (1995). Three genes of the MAP kinase cascade, mek-2, mpk-1/sur-1 and let-60 ras, are required for meiotic cell cycle progression in Caenorhabditis elegans.. Development.

[9] Lee MH, Ohmachi M, Arur S, Nayak S, Francis R (2007). Multiple functions and dynamic activation of MPK-1 extracellular signal-regulated kinase signaling in Caenorhabditis elegans germline development.. Genetics.

[10] Gumienny TL, Lambie E, Hartwieg E, Horvitz HR, Hengartner MO (1999). Genetic control of programmed cell death in the Caenorhabditis elegans hermaphrodite germline.. Development.

[11] Jaramillo-Lambert A, Ellefson M, Villeneuve AM, Engebrecht J (2007). Differential timing of S phases, X chromosome replication, and meiotic prophase in the C. elegans germ line.. Dev Biol.

[12] Miller MA, Nguyen VQ, Lee MH, Kosinski M, Schedl T (2001). A sperm cytoskeletal protein that signals oocyte meiotic maturation and ovulation.. Science.

[13] Sulston JE, Horvitz HR (1977). Post-embryonic cell lineages of the nematode, Caenorhabditis elegans.. Dev Biol.

[14] Sulston JE, Schierenberg E, White JG, Thomson JN (1983). The embryonic cell lineage of the nematode Caenorhabditis elegans.. Dev Biol.

[15] Yan N, Chai J, Lee ES, Gu L, Liu Q (2005). Structure of the CED-4-CED-9 complex provides insights into programmed cell death in Caenorhabditis elegans.. Nature.

[16] Wu D, Wallen HD, Inohara N, Nunez G (1997). Interaction and regulation of the Caenorhabditis elegans death protease CED-3 by CED-4 and CED-9.. J Biol Chem.

[17] Stergiou L, Hengartner MO (2004). Death and more: DNA damage response pathways in the nematode C. elegans.. Cell Death Differ.

[18] Hofmann ER, Milstein S, Boulton SJ, Ye M, Hofmann JJ (2002). Caenorhabditis elegans HUS-1 is a DNA damage checkpoint protein required for genome stability and EGL-1-mediated apoptosis.. Curr Biol.

[19] Schumacher B, Hanazawa M, Lee MH, Nayak S, Volkmann K (2005). Translational repression of C. elegans p53 by GLD-1 regulates DNA damage-induced apoptosis.. Cell.

[20] Bhalla N, Dernburg AF (2005). A conserved checkpoint monitors meiotic chromosome synapsis in Caenorhabditis elegans.. Science.

[21] Salinas LS, Maldonado E, Navarro RE (2006). Stress-induced germ cell apoptosis by a p53 independent pathway in Caenorhabditis elegans.. Cell Death Differ.

[22] Hoole D, Williams GT (2004). The role of apoptosis in non-mammalian host-parasite relationships.. Symp Soc Exp Biol.

[23] Hughes SE, Evason K, Xiong C, Kornfeld K (2007). Genetic and pharmacological factors that influence reproductive aging in nematodes.. PLoS Genet.

[24] Schedl T, Kimble J (1988). fog-2, a germ-line-specific sex determination gene required for hermaphrodite spermatogenesis in Caenorhabditis elegans.. Genetics.

[25] Brenner S (1974). The genetics of Caenorhabditis elegans.. Genetics.

[26] Hedgecock EM, Sulston JE, Thomson JN (1983). Mutations affecting programmed cell deaths in the nematode Caenorhabditis elegans.. Science.

[27] Barton MK, Kimble J (1990). fog-1, a regulatory gene required for specification of spermatogenesis in the germ line of Caenorhabditis elegans.. Genetics.

[28] Yuan J, Horvitz HR (1992). The Caenorhabditis elegans cell death gene ced-4 encodes a novel protein and is expressed during the period of extensive programmed cell death.. Development.

[29] Ellis RE, Jacobson DM, Horvitz HR (1991). Genes required for the engulfment of cell corpses during programmed cell death in Caenorhabditis elegans.. Genetics.

[30] Hengartner MO, Ellis RE, Horvitz HR (1992). Caenorhabditis elegans gene ced-9 protects cells from programmed cell death.. Nature.

[31] Shaham S, Reddien PW, Davies B, Horvitz HR (1999). Mutational analysis of the Caenorhabditis elegans cell-death gene ced-3.. Genetics.

[32] Ellis HM, Horvitz HR (1986). Genetic control of programmed cell death in the nematode C. elegans.. Cell.

[33] Conradt B, Horvitz HR (1998). The C. elegans protein EGL-1 is required for programmed cell death and interacts with the Bcl-2-like protein CED-9.. Cell.

[34] Avery L, Horvitz HR (1987). A cell that dies during wild-type C. elegans development can function as a neuron in a ced-3 mutant.. Cell.

[35] Garigan D, Hsu AL, Fraser AG, Kamath RS, Ahringer J (2002). Genetic analysis of tissue aging in Caenorhabditis elegans: a role for heat-shock factor and bacterial proliferation.. Genetics.

[36] Curran SP, Ruvkun G (2007). Lifespan regulation by evolutionarily conserved genes essential for viability.. PLoS Genet.

[37] Gartner A, Milstein S, Ahmed S, Hodgkin J, Hengartner MO (2000). A conserved checkpoint pathway mediates DNA damage–induced apoptosis and cell cycle arrest in C. elegans.. Mol Cell.

[38] Djahanbakhch O, Ezzati M, Zosmer A (2007). Reproductive ageing in women.. J Pathol.

[39] Broekmans FJ, Knauff EA, te Velde ER, Macklon NS, Fauser BC (2007). Female reproductive ageing: current knowledge and future trends.. Trends Endocrinol Metab.

[40] Tatone C (2008). Oocyte senescence: a firm link to age-related female subfertility.. Gynecol Endocrinol.

[41] Armstrong DT (2001). Effects of maternal age on oocyte developmental competence.. Theriogenology.

[42] Glibert SF (2000). Developmental Biology, 6th Edition. 6 ed..

[43] Fortune JE, Cushman RA, Wahl CM, Kito S (2000). The primordial to primary follicle transition.. Mol Cell Endocrinol.

[44] Matova N, Cooley L (2001). Comparative aspects of animal oogenesis.. Dev Biol.

[45] Peters H, Byskov AG, Grinsted J (1978). Follicular growth in fetal and prepubertal ovaries of humans and other primates.. Clin Endocrinol Metab.

[46] Pepling ME, de Cuevas M, Spradling AC (1999). Germline cysts: a conserved phase of germ cell development?. Trends Cell Biol.

[47] Hsueh AJ, Billig H, Tsafriri A (1994). Ovarian follicle atresia: a hormonally controlled apoptotic process.. Endocr Rev.

[48] McCarter J, Bartlett B, Dang T, Schedl T (1997). Soma-germ cell interactions in Caenorhabditis elegans: multiple events of hermaphrodite germline development require the somatic sheath and spermathecal lineages.. Dev Biol.

[49] Jones TM, Elgar MA (2004). The role of male age, sperm age and mating history on fecundity and fertilization success in the hide beetle.. Proc Biol Sci.

[50] Ng KK, Donat R, Chan L, Lalak A, Di Pierro I (2004). Sperm output of older men.. Hum Reprod.

[51] Yang JW, Lei ZL, Miao YL, Huang JC, Shi LH (2007). Spindle assembly in the absence of chromosomes in mouse oocytes.. Reproduction.

[52] Croen LA, Najjar DV, Fireman B, Grether JK (2007). Maternal and paternal age and risk of autism spectrum disorders.. Arch Pediatr Adolesc Med.

[53] Newschaffer CJ, Croen LA, Daniels J, Giarelli E, Grether JK (2007). The epidemiology of autism spectrum disorders.. Annu Rev Public Health.

[54] Hsin H, Kenyon C (1999). Signals from the reproductive system regulate the lifespan of C. elegans.. Nature.

[55] Kenyon C, Chang J, Gensch E, Rudner A, Tabtiang R (1993). A C. elegans mutant that lives twice as long as wild type.. Nature.

[56] Weir BJ, Rowlands JW, Zuckerman S, Weir BJ (1977). Ovulation and Atresia.. The Ovary.

[57] Baum JS, St George JP, McCall K (2005). Programmed cell death in the germline.. Semin Cell Dev Biol.

[58] Schedl T, Riddle DL, Blumenthal T, Meyer BJ, Priess JR (1997). Developmental Genetics of the Germ Line.. C elegans II.

[59] Cavaliere V, Taddei C, Gargiulo G (1998). Apoptosis of nurse cells at the late stages of oogenesis of Drosophila melanogaster.. Dev Genes Evol.

[60] Baudat F, Manova K, Yuen JP, Jasin M, Keeney S (2000). Chromosome synapsis defects and sexually dimorphic meiotic progression in mice lacking Spo11.. Mol Cell.

[61] Di Giacomo M, Barchi M, Baudat F, Edelmann W, Keeney S (2005). Distinct DNA-damage-dependent and -independent responses drive the loss of oocytes in recombination-defective mouse mutants.. Proc Natl Acad Sci U S A.

[62] Henderson SA, Edwards RG (1968). Chiasma frequency and maternal age in mammals.. Nature.

[63] Wolke U, Jezuit EA, Priess JR (2007). Actin-dependent cytoplasmic streaming in C. elegans oogenesis.. Development.

[64] Lee GD, Wilson MA, Zhu M, Wolkow CA, de Cabo R (2006). Dietary deprivation extends lifespan in Caenorhabditis elegans.. Aging Cell.

[65] Aballay A, Drenkard E, Hilbun LR, Ausubel FM (2003). Caenorhabditis elegans innate immune response triggered by Salmonella enterica requires intact LPS and is mediated by a MAPK signaling pathway.. Curr Biol.

[66] Larsen PL, Albert PS, Riddle DL (1995). Genes that regulate both development and longevity in Caenorhabditis elegans.. Genetics.

[67] Perez GI, Robles R, Knudson CM, Flaws JA, Korsmeyer SJ (1999). Prolongation of ovarian lifespan into advanced chronological age by Bax-deficiency.. Nat Genet.

[68] Gems D, Sutton AJ, Sundermeyer ML, Albert PS, King KV (1998). Two pleiotropic classes of daf-2 mutation affect larval arrest, adult behavior, reproduction and longevity in Caenorhabditis elegans.. Genetics.

[69] Golden TR, Melov S (2007). Gene expression associated with aging in C. elegans. Wormbook..

