# Supplementary material for: Apoptosis Maintains Oocyte Quality in Aging Caenorhabditis elegans Females
Source: PLoS Genet. 2008 Dec 5;4(12):e1000295. doi: 10.1371/journal.pgen.1000295 (PMC2585808; doi:10.1371/journal.pgen.1000295)
Supplement: Dataset S1 — Supporting dataset. (0.13 MB DOC) [file pgen.1000295.s001.doc]

| Figure 1A: | 0 hours aged | |
| --- | --- | --- |
| female x male | n (eggs) | n (mothers) |
| N2 hermaphrodites | 1937 | 7 |
| N2 x q71 | 1479 | 2 |
| N2 x q71 (4 days) | 1077 | 2 |
| q71 x q71 | 5000 | 13 |
| q71 x q71 (4 days) | 5645 | 19 |
| q250 x q71 | 3027 | 3 |
| q250 x q71 (4 days) | 2178 | 3 |

| Figure 1B,C: | 0 hours aged | | 24 hours aged | | 72 hours aged | | 144 hours aged | |
| --- | --- | --- | --- | --- | --- | --- | --- | --- |
| female x male | n (eggs) | n (mothers) | n (eggs) | n (mothers) | n (eggs) | n (mothers) | n (eggs) | n (mothers) |
| q71 x q71 | 5000 | 13 | 7792 | 17 | 3230 | 19 | 1152 | 17 |
| q250 x q71 | 3027 | 3 | 3157 | 7 | 2012 | 8 | 1447 | 16 |

| Figure 3A: | 0 hours | | |
| --- | --- | --- | --- |
| female x male | n (eggs) | n (arrested larvae) | n (mothers) |
| wt x wt | 5000 | 8 | 19 |
| wt x n718 | 1977 | 17 | 4 |
| n718 x wt | 1787 | 21 | 6 |
| n718 x n718 | 2500 | 91 | 7 |
| wt x wt | 5000 | 8 | 19 |
| wt x n1162 | 2097 | 6 | 6 |
| n1162 x wt | 4133 | 20 | 12 |
| n1162 x n1162 | 3269 | 568 | 8 |
| wt x wt | 5000 | 8 | 19 |
| wt x n1950 | 2393 | 111 | 3 |
| n1950 x wt | 3117 | 119 | 4 |
| n1950 x n1950 | 1781 | 276 | 3 |

| Figure 4: | 0 hours aged | | 24 hours aged | | 72 hours aged | | 144 hours aged | |
| --- | --- | --- | --- | --- | --- | --- | --- | --- |
| female x male | n (eggs) | n (mothers) | n (eggs) | n (mothers) | n (eggs) | n (mothers) | n (eggs) | n (mothers) |
| wt x wt | 5000 | 13 | 7792 | 17 | 3230 | 19 | 1152 | 17 |
| wt x n718 | 1977 | 4 | 3532 | 7 | 3388 | 15 | 692 | 8 |
| n718 x wt | 1787 | 6 | 3017 | 7 | 1337 | 16 | 197 | 9 |
| n718 x n718 | 2500 | 7 | 3342 | 9 | 1321 | 17 | 501 | 14 |
| wt x wt | 5000 | 13 | 7792 | 17 | 3230 | 19 | 1152 | 17 |
| wt x n2921 | 1852 | 5 | 2797 | 5 | 2538 | 9 | 194 | 8 |
| n2921 x wt | 2114 | 8 | 1893 | 6 | 1722 | 7 | 257 | 16 |
| n2921 x n2921 | 1818 | 5 | 1909 | 6 | 2183 | 7 | 610 | 17 |
| wt x wt | 5000 | 13 | 7792 | 17 | 3230 | 19 | 1152 | 17 |
| wt x n2439 | 1939 | 3 | 3996 | 14 | 2383 | 12 | 255 | 3 |
| n2439 x wt | 1412 | 4 | 1820 | 5 | 2170 | 18 | 267 | 11 |
| n2439 x n2439 | 1279 | 3 | 996 | 3 | 1597 | 17 | 273 | 10 |
| wt x wt | 5000 | 13 | 7792 | 17 | 3230 | 19 | 1152 | 17 |
| wt x n1162 | 2097 | 6 | 3281 | 9 | 1772 | 13 | 659 | 10 |
| n1162 x wt | 4133 | 12 | 3396 | 9 | 1238 | 12 | 415 | 17 |
| n1162 x n1162 | 3269 | 8 | 2489 | 7 | 1329 | 19 | 380 | 17 |
| wt x wt | 5000 | 13 | 7792 | 17 | 3230 | 19 | 1152 | 17 |
| wt x n2274 | 1735 | 4 | 4272 | 13 | 1688 | 16 | 614 | 8 |
| n2274 x wt | 2613 | 7 | 2167 | 6 | 1060 | 13 | 301 | 17 |
| n2274 x n2274 | 2380 | 6 | 2041 | 7 | 853 | 14 | 340 | 11 |

| Figure 6A: | 0 hours aged | | 24 hours aged | | 72 hours aged | | 144 hours aged | |
| --- | --- | --- | --- | --- | --- | --- | --- | --- |
| female x male | n (eggs) | n (mothers) | n (eggs) | n (mothers) | n (eggs) | n (mothers) | n (eggs) | n (mothers) |
| wt x wt | 5000 | 13 | 7792 | 17 | 3230 | 19 | 1152 | 17 |
| wt x n1084n3082 | 2848 | 4 | 3284 | 8 | 1598 | 13 | 392 | 8 |
| n1084n3082 x wt | 2131 | 7 | 3027 | 8 | 1816 | 7 | 1138 | 14 |
| n1084n3082 x n1084n3082 | 2568 | 7 | 2317 | 7 | 1004 | 5 | 1585 | 13 |
| wt x wt | 5000 | 13 | 7792 | 17 | 3230 | 19 | 1152 | 17 |
| wt x n3330 | 1438 | 2 | 4013 | 7 | 2046 | 11 | 637 | 11 |
| n3330 x wt | 1660 | 3 | 2996 | 6 | 3271 | 11 | 1794 | 12 |
| n3330 x n3330 | 3979 | 6 | 2756 | 6 | 2334 | 10 | 2643 | 12 |
| wt x wt | 5000 | 13 | 7792 | 17 | 3230 | 19 | 1152 | 17 |
| wt x n1950 | 2393 | 3 | 3012 | 9 | 2499 | 15 | 588 | 7 |
| n1950 x wt | 3117 | 4 | 2784 | 6 | 1990 | 10 | 659 | 10 |
| n1950 x n1950 | 1781 | 3 | 3201 | 6 | 1808 | 10 | 583 | 13 |
| wt x wt | 5000 | 13 | 7792 | 17 | 3230 | 19 | 1152 | 17 |
| wt x n1813 | 3315 | 6 | 4603 | 8 | 1078 | 7 | 211 | 6 |
| n1813 x wt | 3630 | 8 | 3934 | 9 | 702 | 6 | 178 | 10 |
| n1813 x n1813 | 2337 | 6 | 2891 | 7 | 755 | 6 | 210 | 9 |
| wt x wt | 5000 | 13 | 7792 | 17 | 3230 | 19 | 1152 | 17 |
| wt x e1735 | 229 | 1 | 5209 | 14 | 2870 | 9 | 752 | 9 |
| e1735 x wt | 7373 | 9 | 978 | 3 | 1835 | 5 | 105 | 8 |
| e1735 x e1735 | 5541 | 9 | 2391 | 6 | 2049 | 9 | 179 | 7 |
| wt x wt | 5000 | 13 | 7792 | 17 | 3230 | 19 | 1152 | 17 |
| all ced-3 females: | 10910 | 33 | 12977 | 36 | 10330 | 82 | 2105 | 77 |
| all ced-4 females: | 12395 | 33 | 10093 | 29 | 4480 | 58 | 1436 | 62 |
